# Risk of thrombotic events and other complications in anticoagulant users infected with SARS-CoV-2: an observational cohort study in primary health care in SIDIAP (Catalonia, Spain)

**DOI:** 10.1186/s12875-022-01752-5

**Published:** 2022-06-08

**Authors:** Maria Giner-Soriano, Ainhoa Gomez-Lumbreras, Cristina Vedia, Dan Ouchi, Rosa Morros

**Affiliations:** 1 Fundació Institut Universitari per a la Recerca a L’Atenció Primària de Salut Jordi Gol i Gurina (IDIAPJGol), Gran Via de les Corts Catalanes 587, àtic. 08007, Barcelona, Spain; 2 Universitat Autònoma de Barcelona, Bellaterra, Cerdanyola del Vallès, Spain; 3 Unitat de Farmàcia, Servei d’Atenció Primària Barcelonès Nord i Maresme, Institut Català de la Salut, Badalona, Spain; 4 Departament de Farmacologia, Terapèutica i Toxicologia. Universitat Autònoma de Barcelona, Bellaterra, Cerdanyola del Vallès, Spain; 5 Institut Català de la Salut, Barcelona, Spain; 6 Plataforma SCReN, UICEC IDIAP Jordi Gol, Barcelona, Spain

**Keywords:** COVID-19, Oral anticoagulants, Thrombotic events, Primary health care, Electronic health records

## Abstract

**Background:**

The risk of thromboembolic events and COVID-19 complications in anticoagulated patients once hospitalized has been widely analyzed. We aim to assess these outcomes in primary health care (PHC) patients chronically treated with oral anticoagulants (OAC) in comparison with non-treated.

**Methods:**

Cohort study including adults with COVID-19 diagnosis in the PHC records in Catalonia, Spain; from March to June 2020. Patients were matched between exposed and non-exposed to OAC based on age and gender in a 1:2 design. Data source is the Information System for Research in Primary Care (SIDIAP).

**Results:**

We included 311,542 individuals with COVID-19. After propensity score matching, we obtained a cohort of 20,360 people, 10,180 exposed and 10,180 non-exposed to OAC. Their mean age was 79.9 and 52.1% were women. Patients exposed to OAC had a higher frequency of comorbidities than non-exposed. Anticoagulated patients had a higher risk of hospital admission (IRR 1.16, 95% CI 1.03–1.29), and of stroke and pulmonary embolism than non-anticoagulated (IRR 1,80, 95% CI 1.06–3.06). The risk of pneumonia was not different between groups (IRR 1.04, 95% CI 0.84–1.30). We found a lower risk of death in patients exposed to OAC (IRR 0.60, 95% CI 0.55–0.65).

**Conclusions:**

OAC users in our study had more comorbidities and were older than non-users, well known risks for hospitalization being confirmed with our results. We also found in our study that OAC exposure was not associated to an increased risk in the mortality rate, and it was associated with higher risks of hospital admission and thromboembolic events, although we cannot assess the effect of the interventions applied during hospital admission on the outcomes studied, as our database is a PHC database.

**Trial registration:**

EUPAS register: EUPAS37205.

**Supplementary Information:**

The online version contains supplementary material available at 10.1186/s12875-022-01752-5.

## Background

Coronavirus disease 2019 (COVID-19) is a viral respiratory illness caused by the severe acute respiratory syndrome-coronavirus-2 (SARS-CoV-2), emerged as a global public health crisis in 2020 [1–3]. Previous research has highlighted that patients with prior cardiovascular conditions are at higher risk for adverse outcomes from COVID-19 [4] and also may predispose patients to thrombotic events [5–7] due to both direct and indirect effects of infection, such as severe illness, hypoxia or hypercoagulability [5, 8, 9].

On the one hand, anticoagulant therapy, as low-molecular weight heparins (LMWH) or oral anticoagulants (OAC), is routinely used to treat thrombotic complications in COVID-19 patients admitted to hospital [10–12]. On the other hand, patients who are chronically anticoagulated for conditions such as atrial fibrillation are at higher risk of thrombotic events [4], and guidelines have previously established the considerations on the use of OAC in COVID-19 patients [10, 13]. Ambulatory patients already on OAC could then be thought of having less risk for thrombosis event during the COVID-19 infection thought the effects of OAC in the occurrence and prognosis of complications of SARS-CoV-2 in patients chronically treated with OAC are still unknown, as little data are currently available on the prognosis and risk factors of patients exposed to OAC prior to COVID-19 infection [5, 14].

Most studies are focused on analyzing the risk of thromboembolic events and complications from the COVID-19 infection in patients on anticoagulant treatment once hospitalized [15–17].

We aimed to assess these outcomes in primary health care (PHC) patients who are chronically treated with OAC in comparison with a group of non-treated with OAC.

## Methods

### Study design

Cohort study including adult patients with COVID-19 diagnosis registered as confirmed (by polymerase chain reaction, PCR) or as probably (not confirmed by PCR) in the PHC records in Catalonia, Spain; from the pandemics’ onset (March 2020) to June 30^th^, 2020. Patients were followed up from study inclusion up to the end of June 2020.

### Data source

The study data source is the Information System for Research in Primary Care (SIDIAP) [18], which captures clinical information of approximately 5,8 million people from Catalonia, Spain (around 80% of the Catalan population). This information is pseudonymized, originated from different data sources: 1) ECAP (electronic health records in PHC of the Catalan Health Institute); including socio-demographic characteristics, residents in nursing homes/long-term facilities (LTCF), comorbidities registered as International Classification of Disease (ICD)-10 codes [19], specialist referrals, clinical parameters, toxic habits, sickness leave, date of death, laboratory test data, and drug prescriptions issued in PHC, registered as chemical classification system (ATC) codes [20]; 2) pharmacy invoice data corresponding to the PHC drug prescriptions; 3) database of diagnoses at hospital discharge [21] and 4) COVID-19 data from the Catalan Agency of Health Quality and Evaluation (AQuAS) [22].

### COVID-19 classification

Subjects were classified according to the following criteria: confirmed cases are those with a confirmed COVID-19 diagnostic record, PCR + and/or a positive serology test. Those with a non-confirmed diagnosis or test (possible or unclear) along with any individual with a record of hospitalization, pneumonia and/or death related to COVID-19 were considered possible cases. During the first wave of the COVID-19 pandemics in Catalonia, PCR tests were not routinely conducted to all patients with compatible symptoms, due to the unavailability of laboratory kits to do the tests. Thus, we needed to capture those patients with possible diagnosis of COVID-19, such as those admitted to hospital with pneumonia or other COVID-19 symptoms who were not tested. We designed an algorithm to classify patients as “COVID possible” when there was not a test result available. The date of COVID-19 diagnosis was set to be the first of all records used per patient.

To guarantee that our algorithm is not far from the Catalan population, the resulting cohort was compared to the official COVID-19 cases provided by the AQuAS during the pandemic (comparison in supplementary material, Figure S1) [22, 23].

### Drug exposure

Patients were classified as exposed to OAC if they had at least one dispensation of either direct OAC (DOAC) or vitamin K antagonists (VKA) during the three previous months to the COVID-19 diagnosis date.

### Variables

At baseline, the variables captured were: sex, age, geographical area, MEDEA socioeconomic index (deprivation index based on five indicators of socio-economic position. The higher this is, the worse the deprivation is, and it allows analyzing health inequalities) [24], body mass index (BMI), residence in nursing homes, smoking habit, comorbidities, and drug exposure to OAC and to comedications which might be associated with the COVID-19 prognosis and/or the events studied: antiplatelets, non-steroidal anti-inflammatory drugs, systemic corticosteroids and LMWH.

Main outcomes: diagnosis of pneumonia, thrombotic composite outcome containing stroke and pulmonary embolism, hospital admission, and mortality. The risk of these events was analyzed comparing patients exposed to OAC with non-exposed.

### Statistical analysis

Quantitative variables were described as the mean and standard deviation, whereas categorical variables were described as the proportion over the exposed and non-exposed individuals. Univariate analysis was done by means of Student’s t-test and Chi-square test as appropriate.

For each outcome, we fitted a negative binomial regression (NB) to estimate the incidence rate ratio (IRR) comparing the incidence of each outcome (event per 1,000 person-day) among those exposed to OAC to those non-exposed to the drug. To better understand the OAC effect on the outcomes, we also reported the IRR on a propensity score-matched population by using the nearest neighbor method with the covariates age, gender, smoking habits, comorbidities, and concomitant drugs. The confidence intervals (95% CI) and *p*-values were derived using robust standard errors at a 0.05 level [25]. All analyses were performed in R software (v3.6.3).

We conducted a sensitivity analysis of the risk of the events excluding those patients living in nursing homes or long-term care facilities.

## Results

Of a cohort of 311,542 individuals with COVID-19 registers, 11,828 (3.8%) had been previously exposed to OAC. The whole cohort was matched by propensity score, resulting in 20,360 individuals, 10,180 exposed and 10,180 non-exposed to OAC. Table 1 includes all baseline sociodemographic and clinical characteristics of patients included in the study before and after the propensity score matching. Patients in the matched cohort were mostly women (52,1%) and with a mean age of 79,9 years-old. The most frequent comorbidities were hypertension (77,1%), respiratory diseases (37%) and diabetes (32.1%).

**Table 1:** Baseline sociodemographic and clinical characteristics of patients included in the study before and after the propensity score matching^b^

	**All COVID-19 patients**			**PS matched COVID-19 patients**		
	**Total**	**Non-exposed to OAC**^e^	**Exposed to OAC**	**Total after PS matching**	**Non-exposed to OAC (matched)**	**Exposed to OAC**
**N (%)**	311,542	299,714	11,828	20,360	10,180	10,180
**Male sex**	137,258 (44.1)	131,466 (43.9)	5792 (49.0)	9748 (47.9)	4869 (47.8)	4879 (47.9)
**Age, mean (SD)**	49.3 (22.3)	48.2 (21.8)	79.3 (11.8)	79.9 (12.3)	80.6 (12.6)	79.2 (12.0)
**Smoking habit**	120,632 (38.7)	115,326 (38.5)	5306 (44.9)	9022 (44.3)	4478 (44.0)	4544 (44.6)
**BMI, mean (SD)**^a^	26.8 (6.3)	26.6 (6.3)	29.1 (5.6)	28.7 (5.5)	28.2 (5.3)	29.2 (5.6)
**Obesity**^a^	86,182 (50.5)	79,789 (49.6)	3199 (33.4)	10,172 (39.2)	6973 (42.7)	3199 (33.4)
**COVID-19 diagnosis**						
*Positive*	164,557 (52.8)	158,958 (53.0)	5599 (47.3)	9456 (46.4)	4941 (48.5)	4515 (44.4)
*Possible*	146,985 (47.2)	140,756 (47.0)	6229 (52.7)	10,904 (53.6)	5239 (51.5)	5665 (55.6)
**LTCF**	28,361 (9.1)	25,221 (8.4)	3140 (26.5)	6524 (32.0)	3657 (35.9)	2867 (28.2)
**MEDEA**						
*Unknown*	84,225 (27.0)	80,384 (26.8)	3841 (32.5)	6877 (33.8)	3627 (35.6)	3250 (31.9)
*Rural area*	57,121 (18.3)	55,050 (18.4)	2071 (17.5)	3851 (18.9)	2028 (19.9)	1823 (17.9)
*Urban quintiles 1–3*	138,373 (44.4)	133,279 (44.5)	5094 (43.0)	8787 (43.2)	4331 (42.5)	4456 (43.8)
*Urban quintiles 4–5*	88,944 (28.5)	86,051 (28.75)	2893 (24.4)	4696 (23.1)	2222 (21.8)	2474 (24.3)
**Comorbidities**^c^						
**Chronic kidney disease**	19,242 (6.2)	15,497 (5.2)	3620 (30.6)	6254 (30.7)	3154 (31.0)	3100 (30.5)
**Diabetes**	31,079 (10.0)	27,198 (9.1)	3883 (32.8)	6539 (32.1)	3259 (32.0)	3280 (32.2)
**Hypertension**	77,413 (24.8)	68,395 (22.8)	8998 (76.1)	15,689 (77.1)	7956 (78.2)	7733 (76.0)
**Heart failure**	9649 (3.1)	5837 (1.9)	3393 (28.7)	5242 (25.7)	2359 (23.2)	2883 (28.3)
**Ischemic coronary disease**	10,261 (3.3)	8246 (2.8)	2044 (17.3)	3187 (15.7)	1466 (14.4)	1721 (16.9)
**Respiratory disease**	79,060 (25.4)	73,444 (24.5)	4460 (37.7)	7528 (37.0)	3747 (36.8)	3781 (37.1)
**Thromboembolism**	1994 (0.6)	1229 (0.4)	589 (5.0)	616 (3.0)	93 (0.9)	523 (5.1)
**Concomitant Drugs**^d^						
**Antiplatelets**	22,137 (7.1)	21,378 (7.1)	759 (6.4)	1417 (7.0)	797 (7.8)	620 (6.1)
**LMWH**	2841 (0.9)	2414 (0.8)	427 (3.6)	755 (3.7)	409 (4.0)	346 (3.4)
**NSAID**	76,224 (24.5)	71,008 (23.7)	5223 (44.2)	8841 (43.4)	4317 (42.4)	4524 (44.4)
**Systemic corticosteroids**	16,138 (5.2)	14,873 (5.0)	1267 (10.7)	2180 (10.7)	1088 (10.7)	1092 (10.7)

^a^BMI > 30 or ICD-10 diagnosis

^b^Obtained from a Chi-square test in categorical variables, and t-test in numerical variables

^c^Starting or active one year before COVID-19 diagnosis

^d^Registered dispensation three months before COVID-19 diagnosis

^e^*OAC* oral anticoagulants, *SD* standard deviation, *BMI* body mass index, *LTCF* long-term care facilities, MEDEA deprivation index based on five indicators of socio-economic position. The higher this is, the worse the deprivation [24]. LMWH; low-molecular weight heparins. NSAID; nonsteroidal anti-inflammatory drugs

The frequencies of events of interest which were assessed after the diagnosis of COVID-19 are shown in Table 2. Patients exposed to OAC showed a higher frequency of stroke and pulmonary embolism and of hospital admission (1% vs 0.6%, 0.8% vs 0.2%, and 7.9% vs 6.9%, respectively) than the non-exposed.

**Table 2:** Number of events of interest after the COVID-19 diagnosis

	**All COVID-19 patients**			**PS matched COVID-19 patients**		
	**Total**	**Non-exposed to OAC**^a^	**Exposed to OAC**	**Total after PSa matching**	**Non-exposed to OAC (matched)**	**Exposed to OAC**
**N (%)**	311,542	299,714	11,828	20,360	10,180	10,180
**Pneumonia**	5751 (1.8)	5421 (1.8)	330 (2.8)	476 (2.3)	234 (2.3)	242 (2.4)
**Stroke**	638 (0.2)	521 (0.2)	117 (1.0)	169 (0.8)	65 (0.6)	104 (1.0)
**Pulmonary embolism**	548 (0.2)	423 (0.1)	125 (1.1)	109 (0.5)	25 (0.2)	84 (0.8)
**Hospital admission**	18,440 (5.9)	16,636 (5.6)	1804 (15.3)	1510 (7.4)	706 (6.9)	804 (7.9)
**Death**	1254 (0.4)	1062 (0.4)	192 (1.6)	145 (0.7)	76 (0.7)	69 (0.7)

^a^*OAC* oral anticoagulants, *PS* propensity score

**Fig. 1:**
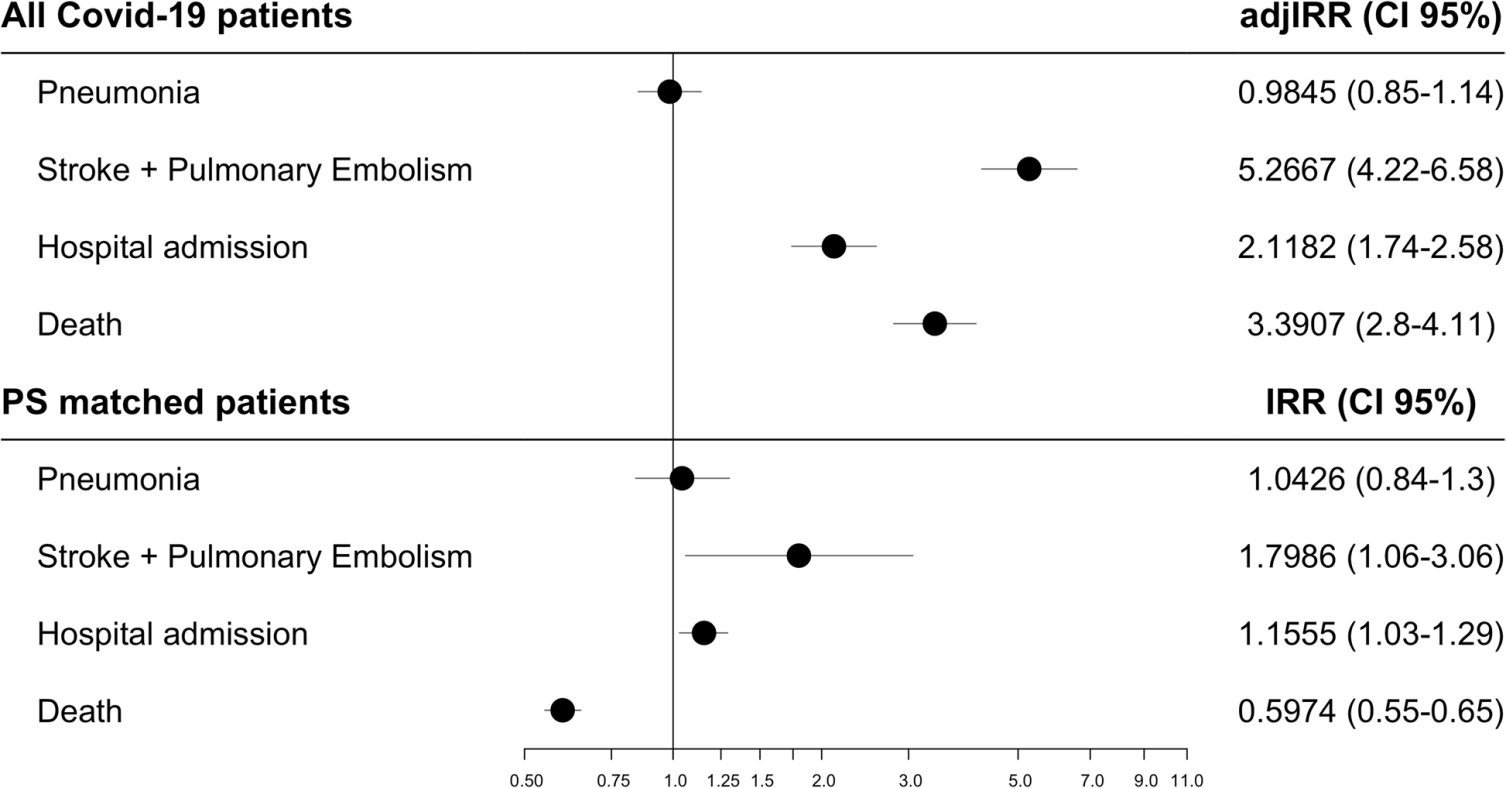
Forest plot incidence rate rations among individuals exposed to oral anticoagulants. IRR; incidence rate ratios. PS; propensity score

The incidence rate for the events of interest per 1000 person-day are shown in Table 3, along with the IRR. The results of the NB regression are shown in Fig. 1. For the propensity score matched patients, those who were exposed to OAC had a higher risk of hospital admission (IRR 1.16, 95% CI 1.03–1.29), and the composite endpoint composed by stroke and pulmonary embolism than non-anticoagulated patients (IRR 1.80, 95% CI 1.06–3.06). The risk of pneumonia was not different between exposure groups (IRR 1.04, 95% CI 0.84–1.30). The risk of death was lower for patients exposed to OAC compared to non-exposed (IRR 0.60, 95% CI 0.55–0.65).

**Table 3:** Incidence rate ratios from the fitted negative binomial regression

	**Non-exposed to OAC**			**Exposed to OAC**								
Outcome	**sum of events**	**sum of person-days**	**Incidence rate (events/1000 person-day)**	**sum of events**	**sum of person-days**	**Incidence rate (events/1000 person-day)**	**IRR**	**95% CI**^**a**^	***P*-value**	**PS matched samples IRR**	**95% CI**^**a**^	***P*-value**
Pneumonia	4577	20,955,070	0.22 (0.21–0.22)	242	696,364	0.35 (0.3–0.39)	0.98	(0.85–1.14)	< .001	1.04	(0.84–1.3)	0.710
Stroke and pulmonary embolism	764	20,955,070	0.04 (0.03–0.04)	188	696,364	0.27 (0.23–0.31)	5.27	(4.22–6.58)	< .001	1.80	(1.06–3.06)	0.030
Hospital admission	9478	20,955,070	0.45 (0.44–0.46)	804	696,364	1.15 (1.08–1.24)	2.12	(1.74–2.58)	< .001	1.16	(1.03–1.29)	0.013
Death	8193	20,955,070	0.39 (0.38–0.4)	1318	696,364	1.89 (1.79–2)	3.39	(2.80–4.11)	< .001	0.60	(0.55–0.65)	< .001

^a^95% CI and *P*-values are derived using robust standard errors as suggested in Long J. S., Ervin L. H. [25]

Adjusted IRR by age, gender, smoking habits, comorbidities and comedications

*OAC* oral anticoagulants, *IRR* incidence rate ratio

## Discussion

We analyzed the thromboembolic and fatal outcomes in a COVID-19 cohort, comparing those individuals who were exposed to OAC to those non-exposed, matched by age and sex. In our study, those patients exposed to OAC had a higher risk of hospital admission and thromboembolic events – stroke and pulmonary embolism– than those who were not treated with OAC. Those patients exposed to OAC had a lower risk of mortality than non-exposed. We found no differences in the risk of pneumonia between groups.

Patients with cardiovascular conditions have been reported as having a higher risk for adverse events from COVID-19 and thrombotic events [4–7]. For instance, Inciardi et al. analyzed data of 99 COVID-19 patients with pneumonia, 53.5% of them had previous cardiovascular diseases, and found a higher mortality in this subgroup of patients (RR 2.35, 95% CI 1.08–5.09). They did not report on the use of comedications [26].

As anticoagulant therapy as LMWH is used to treat thrombotic complications in COVID-19 patients, chronic treatment with OAC may play a role in lowering the risk of thrombotic events caused by SARS-CoV-2 infection, and in this alignment, some studies have previously assessed complications in COVID-19 patients according to their previous exposure to OAC, finding different results. Rivera-Caravaca et al. studied 1,002 patients from Ecuador, Germany, Italy and Spain who were admitted to hospital due to COVID-19 and were previously treated with OAC. They found a HR of 1.53 (CI95% 1.08–2.16) for mortality when compared OAC users with non-users [27].

Russo et al. enrolled 192 COVID-19 patients to study the risk of suffering acute respiratory distress syndrome (ARDS) and/or death during hospitalization. They found no protective effect for these events in the 26 (13.5%) patients who were OAC users admitted to hospital [28].

Denas et al. conducted a population-based propensity score-matched study in the Veneto Region, Italy. They included 4,697 COVID-19 patients older than 65 and matched 559 anticoagulated patients to 559 non-exposed. They found a rate ratio of all-cause mortality higher in non-anticoagulated patients (32.2% vs 26.5%), although the estimate was not statistically significant; HR 0.81, 95%CI 0.65–1.01. The authors discussed a possible role of OAC in reducing the mortality in that group of patients, although taking into account that they might have been switched to LMWH during hospitalization and other interventions might have also affected the outcomes [29]. Similar results, finding OAC exposure as having a protecting role, are shown in an Italian study [HR for death 0.38 (0.17–0.58)] [30]. Our findings do not show a higher risk among those exposed to OAC, risk that also do not show when analyzing a subpopulation of those patients not in long-term facilities.

Tremblay et al. conducted a study in 3,772 COVID-19 patients treated with OAC, antiplatelets or non-treated with antithrombotics. They did two propensity score matchings, comparing OAC-treated vs non-treated and antiplatelets-treated vs non-treated. The HR for all-cause mortality, mechanical ventilation and hospital admission were not significant. The Kaplan-Meyer survival analyses did not show differences between groups. They did not exclude the possibility that anticoagulation during COVID-19 infection may be useful but they did not find that previous treatment with OAC may protect from severe forms of COVID-19 [31].

In a study in France, 90% of COVID-19 patients admitted to the ICU because of pulmonary thromboembolism were receiving prophylactic anticoagulant (LMWH) treatment according to critically ill patients’ guidelines, but the study did not analyze if these patients were already on prophylactic anticoagulant treatment due to previously indications such as atrial fibrillation [32].

We found that OAC users were more frequently hospitalized than non-users. This outcome has not been assessed in the cited studies above, as all patients included in those studies were already hospitalized for SARS-CoV-2 infection [15, 16, 32, 33]. Our study includes those hospitalized and those who not, and it reveals that anticoagulated patients had more comorbidities and were older, well known risks for hospitalization being confirmed with our results.

Among the limitations of our study there is the reliability of the COVID-19 diagnoses; we included patients without a confirmed result as during the first wave of the pandemic in our setting PCR test were not always performed. This limitation has been described in other research as during the beginning of the pandemic diagnosis test for COVID-19 were not widely available, and clinical algorithms have been used to assess COVID-19 diagnosis [34]. Another limitation is the lack of hospital information: we cannot capture ICU admission, ventilation, treatments administered during the admission, or other indicators of the severity of the infection, which clearly have influence in the prognosis and outcomes of COVID-19. A third limitation is the lack of availability of the indication for oral anticoagulation, making impossible to address a potential confounding by indication bias in our study.

We also found in our study that OAC exposure was not associated to an increased risk in the mortality rate, although we cannot assess the effect of the interventions applied during hospital admission on the mortality, scales of prognosis, complications, treatments and specific outcomes in COVID-19 hospital admitted patients, as our database is a PHC database and does only record the dates of admission and diagnoses and cause of discharge.

Some strengths of our study include the large number of patients included, representativeness for general population, and complete socio-demographic data. We must highlight that our cohort are PHC patients, so we have estimated the risk of death and hospitalization for a different population from the only hospitalized ones that are usually studied.

## Conclusions

OAC users in our study had more comorbidities and were older than non-users, well known risks for hospitalization being confirmed with our results.

We found that OAC exposure was not associated to an increased risk in the mortality rate in our study, although we cannot assess the effect of the interventions applied during hospital admission on the COVID-19 outcomes, as our database is a PHC database.

We must highlight that our cohort are PHC patients, so we have estimated the risk of death and hospitalization for a different population from the only hospitalized ones that are usually studied.

## Supplementary Information

**Additional file 1: Figure S1.** COVID-19 cases between March and July 2020 according to Catalan Agency of Health (source: https://dadescovid.cat/?lang=eng) compared to SIDIAP database.

## Data Availability

The datasets generated and/or analysed during the current study are not publicly available due to patient privacy and data protection concerns, but they are available from the corresponding author on reasonable request. All data generated or analysed during this study are included in this published article and its supplementary information files.

## Abbreviations

AQuAS: Catalan Agency of Health Quality and Evaluation
ATC: Anatomical therapeutic chemical classification
BMI: Body mass index
CI: Confidence interval
COVID-19: Coronavirus disease 2019
DOAC: Direct oral anticoagulants
ECAP: Electronic health records in primary care of the Catalan Health Institute
ICD: International classification of diseases
IDIAPJGol: Fundació Institut Universitari per a la recerca a l’Atenció Primària de Salut Jordi Gol i Gurina
IRR: Incidence rate ratio
LMWH: Low-molecular weight heparins
MEDEA: Socioeconomic index
NB: Negative binomial
OAC: Oral anticoagulants
OR: Odds ratio
PCR: Polymerase chain reaction
PHC: Primary health care
PS: Propensity score
SARS-CoV-2: Severe acute respiratory syndrome-coronavirus-2
SD: Standard deviation
SIDIAP: Information system for research in primary care
VKA: Vitamin K antagonists
