# Supplementary material for: Risk of thrombotic events and other complications in anticoagulant users infected with SARS-CoV-2: an observational cohort study in primary health care in SIDIAP (Catalonia, Spain)
Source: BMC Prim Care. 2022 Jun 8;23:147. doi: 10.1186/s12875-022-01752-5 (PMC9174624; doi:10.1186/s12875-022-01752-5)

### Figure S1. COVID-19 cases between March and July 2020 according to Catalan Agency of Health (source: <https://dadescovid.cat/?lang=eng>) compared to SIDIAP database


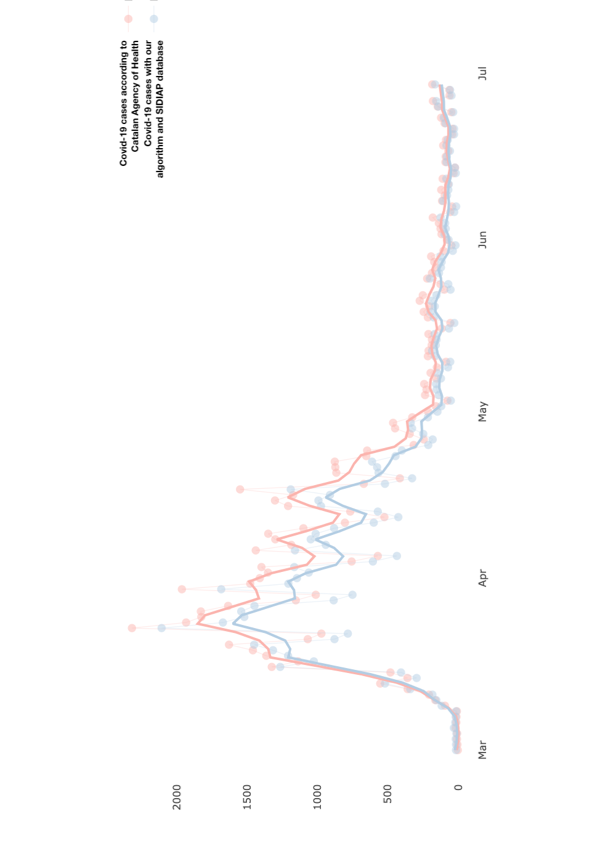

Supplement: Supplementary file 1 — Additional file 1: Figure S1. COVID-19 cases between March and July 2020 according to Catalan Agency of Health (source: https://dadescovid.cat/?lang=eng) compared to SIDIAP database. [file 12875_2022_1752_MOESM1_ESM.docx]
